# Recombinant Human Plasma Gelsolin Stimulates Phagocytosis while Diminishing Excessive Inflammatory Responses in Mice with *Pseudomonas aeruginosa* Sepsis

**DOI:** 10.3390/ijms21072551

**Published:** 2020-04-07

**Authors:** Ewelina Piktel, Urszula Wnorowska, Mateusz Cieśluk, Piotr Deptuła, Suhanya V. Prasad, Grzegorz Król, Bonita Durnaś, Andrzej Namiot, Karolina H. Markiewicz, Katarzyna Niemirowicz-Laskowska, Agnieszka Z. Wilczewska, Paul A. Janmey, Joanna Reszeć, Robert Bucki

**Affiliations:** 1 Department of Medical Microbiology and Nanobiomedical Engineering, Medical University of Bialystok, Mickiewicza 2c, 15-222 Bialystok, Poland; ewelina.piktel@wp.pl (E.P.); u.wnorowska@gmail.com (U.W.); mticv1@gmail.com (M.C.); piotr.deptula@umb.edu.pl (P.D.); suhanyavp@gmail.com (S.V.P.); katia146@wp.pl (K.N.-L.); 2 Department of Microbiology and Immunology, the Faculty of Medicine and Health Sciences of the Jan Kochanowski University in Kielce, Stefana Żeromskiego 5, 25-001 Kielce, Poland; g.krol@op.pl (G.K.); bonita.durnas@onkol.kielce.pl (B.D.); 3 Department of Anatomy, Medical University of Bialystok, Mickiewicza 2b, 15-222 Bialystok, Poland; anamiot@poczta.onet.pl; 4 Institute of Chemistry, University of Białystok, Ciołkowskiego 1K, 15-245 Bialystok, Poland; k.markiewicz@uwb.edu.pl (K.H.M.); agawilczuwb@gmail.com (A.Z.W.); 5 Institute for Medicine and Engineering, University of Pennsylvania, 3340 Smith Walk, Philadelphia, PA 19104, USA; janmey@pennmedicine.upenn.edu; 6 Department of Pathology, Medical University of Bialystok, Waszyngtona 13, 15-269 Bialystok, Poland; joannareszec@gmail.com

**Keywords:** plasma gelsolin, inflammation, phagocytosis, sepsis, *Pseudomonas aeruginosa*

## Abstract

Plasma gelsolin (pGSN) is a highly conserved abundant circulating protein, characterized by diverse immunomodulatory activities including macrophage activation and the ability to neutralize pro-inflammatory molecules produced by the host and pathogen. Using a murine model of Gram-negative sepsis initiated by the peritoneal instillation of *Pseudomonas aeruginosa* Xen 5, we observed a decrease in the tissue uptake of IRDye^®^800CW 2-deoxyglucose, an indicator of inflammation, and a decrease in bacterial growth from ascitic fluid in mice treated with intravenous recombinant human plasma gelsolin (pGSN) compared to the control vehicle. Pretreatment of the murine macrophage line RAW264.7 with pGSN, followed by addition of *Pseudomonas aeruginosa* Xen 5, resulted in a dose-dependent increase in the proportion of macrophages with internalized bacteria. This increased uptake was less pronounced when cells were pretreated with pGSN and then centrifuged to remove unbound pGSN before addition of bacteria to macrophages. These observations suggest that recombinant plasma gelsolin can modulate the inflammatory response while at the same time augmenting host antibacterial activity.

## 1. Introduction

Plasma gelsolin (pGSN) is a multifunctional, actin-binding protein involved in a number of physiological and pathological processes including, but not limited to, the removal of actin filaments from the bloodstream leaked from dead cells as the result of tissue injury [1,2]. Recently, pGSN was recognized as a key factor modulating host immune responses due to the preferential binding of microbial-derived endotoxins, resulting in prevention of toll-like receptor (TLR) activation [3,4]. A compelling number of studies also recognize plasma gelsolin as a potential biomarker of inflammation [5,6,7,8,9,10,11].

It is suggested that repletion with exogenous recombinant pGSN might preempt, limit, or reverse the negative effects of hypogelsolinemia [5]. The pleotropic actions of pGSN supplementation could improve the host antimicrobial defense by different mechanisms, including the improvement of bacterial clearance by macrophages via nitric oxide synthase 3 (NOS3)-dependent mechanisms and restoring bacterial binding and uptake by murine and human macrophages, impaired during acute tissue injuries [4,12]. A direct bactericidal action of pGSN has not been demonstrated so far [13]. On the other hand, injections of recombinant pGSN improve survival and clinical outcomes in septic mice subjected to lethal doses of LPS or polymicrobial challenge after cecal ligation and puncture (CLP). Its beneficial effect can in part be attributed to its broad-spectrum anti-inflammatory buffering and altering the inflammatory cytokine expression [12,14]. The cumulative results support beneficial effects of gelsolin on host immune and inflammatory processes. Nevertheless, there is still limited data about the biodistribution and elimination of exogenously administrated pGSN and its effect on promoting phagocytosis while limiting overzealous and injurious inflammatory processes.

In this study, we characterized the therapeutic efficiency of intravenously administrated pGSN in an animal model of sepsis as a consequence of peritonitis induced by *P. aeruginosa* Xen5, a bioluminescent strain derived from a human septicemia isolate. pGSN biodistribution after exogenous administration was also elucidated. This study shows that exogenous pGSN stimulates host antibacterial mechanisms, including phagocytosis, which curtail inflammatory responses to a bacterial challenge.

## 2. Results

### 2.1. pGSN-800CW Synthesis and Physicochemical Evaluation of Dye-Labeled pGSN

To assess the biodistribution of pGSN and, in further experiments, inflammation severity in tested animals, we used agents labeled with IRDye^®^ 800CW, a fluorescent dye, which is detected by the 800 nm channel using a Pearl^®^ Trilogy small animal imaging system. To date, a few studies were performed to elucidate the pharmacokinetic and toxicity of intravenously administrated IRDye 800CW [15,16]. According to Marshall et al. the clearance half-time of IRDye 800CW following intravenous (i.v.) injection is 35.7 min, which indicates that the dye is rapidly eliminated from the body [15]. Moreover, tissue section analyses performed by Kovar et al. demonstrated that the fluorescence signal recorded for organs from IRDye 800CW-injected animals was only slightly higher than the signal obtained for PBS-treated animals [16].

For the purpose of this study, the formation of a stable conjugate of pGSN with fluorescent IRDye^®^ 800CW was performed by ester-mediated derivatization by coupling free amino groups of pGSN to the N-hydroxysuccinimide (NHS) reactive group present in IRDye^®^ 800CW [17]. In order to confirm the proper dye conjugation with pGSN, FTIR spectroscopy and thermal analysis, before and after labelling were performed (Figure S1). According to Figure S1A, the IR spectrum of pGSN- 800CW conjugate shows significant changes compared to unmodified pGSN, primarily in the fingerprint region. The stretching vibrations of N-H (~3263 cm^−1^), C-H (2965–2850 cm^−1^), C=O (amide I, ~1630 cm^−1^) and N-H (amide II, ~1540 cm^−1^) groups are present in both spectra. Several new absorption bands appeared in the pGSN-800CW spectrum at 1406 cm^−1^, 1327 cm^−1^ (=CH); 1150 cm^−1^, 1102 cm^−1^ (SO_3_^−^); 1074 cm^−1^, 1030 cm^−1^ (Ar-O-C); and 987 cm^−1^ (Ar-H), confirming protein modification. The labelled protein was also characterized by differential scanning calorimetry (DSC) and thermogravimetric analysis (TGA) (Figure S1B–D). The heating curves of pGSN before and after functionalization indicate differences in the chemical structure of these compounds (Figure S1B). A large endothermic transition is observed in the temperature range from 250 to 350 °C. It is most likely associated with the presence of the dye molecule and its decomposition. Panels C and D in Figure S1 show TGA results for pGSN and pGSN-800CW conjugate. DSC profiles of the IRDye^®^ 800CW-conjugated pGSN and the unlabeled protein indicate successful functionalization, with ~5% IRDye^®^ 800CW content.

### 2.2. The Biodistribution of IRDye^®^ 800CW-Labeled pGSN in Healthy and PA Xen5-Infected Mice

Before starting the experiments, animals were divided into six groups, as indicated in Table 1.

**Figure 1:**
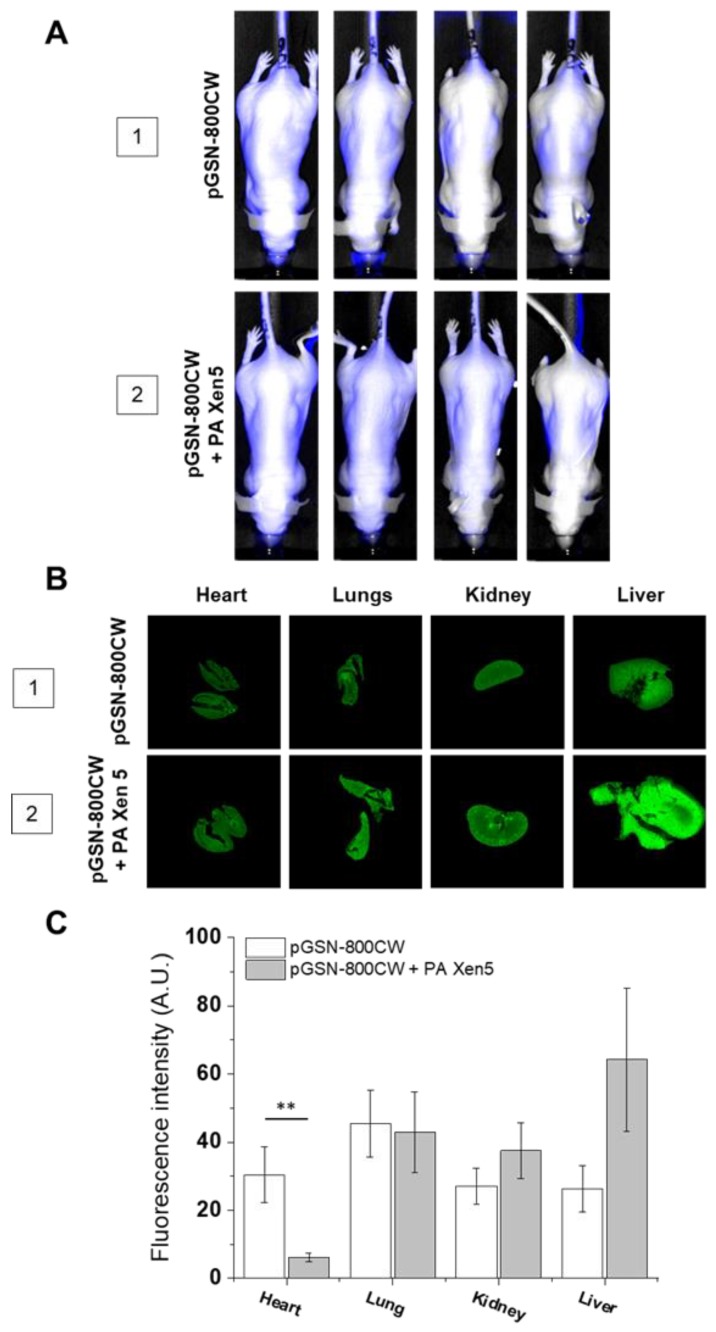
Biodistribution of exogenously administrated pGSN labelled with IRDye^®^800CW (pGSN-800CW) estimated by fluorescence-based analysis of pGSN-800CW-targeted fluorescence signal in Cby.Cg-Foxn1nu/cmdb mice. At the start of the experiment, mice were injected intravenously with sterile saline (healthy mice; group 1) or PA Xen5 inoculum (infected mice; group 2), 8 h later IRDye 800CW-labeled pGSN was added (40 mg/kg) to both groups of animals. After sacrificing the animals (24 h after injection of pGSN-800CW), the hearts, lung, kidney and livers were collected and fluorescence signal was registered at 800 nm channel. Whole mouse scans representing distribution of labeled plasma gelsolin were performed upon the injection of pGSN-800 CW (40 mg/kg) into healthy (group 1) and PA Xen5-infected animals (group 2). Data were collected after 0.5, 4, 8 and 24 h from starting of observation. Results from representative animals are shown (**A**). Representative scans of organs collected from sacrificed healthy (group 1) and Pa Xen5-infected animals (group 2) (**B**). Organ uptake of fluorophore-labeled plasma gelsolin was estimated based on fluorescence intensity of collected organs and presented as mean value ± SEM from three areas of each organ. ** indicates statistical significance (*p* < 0.01; assessed using two-tailed Student’s *t*-test) when comparing to organs in non-infected animals (**C**).

IRDye^®^ 800CW-labeled pGSN allowed for in vivo imaging of gelsolin biodistribution, both in healthy and bacteria-challenged animals (Figure 1). At 24 h post-injection the highest accumulation of pGSN in healthy mice was observed in lungs, and smaller amounts were evenly distributed in the heart, kidneys, and liver. In bacteria-infected animals, the distribution profile was altered considerably. A statistically significant decrease in pGSN accumulation was observed in the hearts of sacrificed animals. At the same time, higher pGSN levels were noted in kidney and liver, but this effect did not reach statistical significance. At the same time, lung accumulation of pGSN remained at the same level.

### 2.3. Administration of pGSN Decreases the Inflammatory Response in Septic Mice

According to previously published records, human plasma gelsolin circulates in blood at concentrations of 150–300 μg/mL, with some variations depending on the detection method employed [18,19,20]. Considering the reports indicating the accelerated clearance of pGSN when complexed with extracellular actin, which is recognized as a crucial mechanism resulting in rapid decrease of pGSN level in patients with sepsis and systemic inflammation [20], we decided to use a dose of gelsolin slightly exceeding the physiological concentration (~400 µg/mL or 40 mg/kg) observed in human blood, to maintain the appropriate concentration of recombinant protein throughout the duration of treatment, i.e., 24 h.

**Figure 2:**
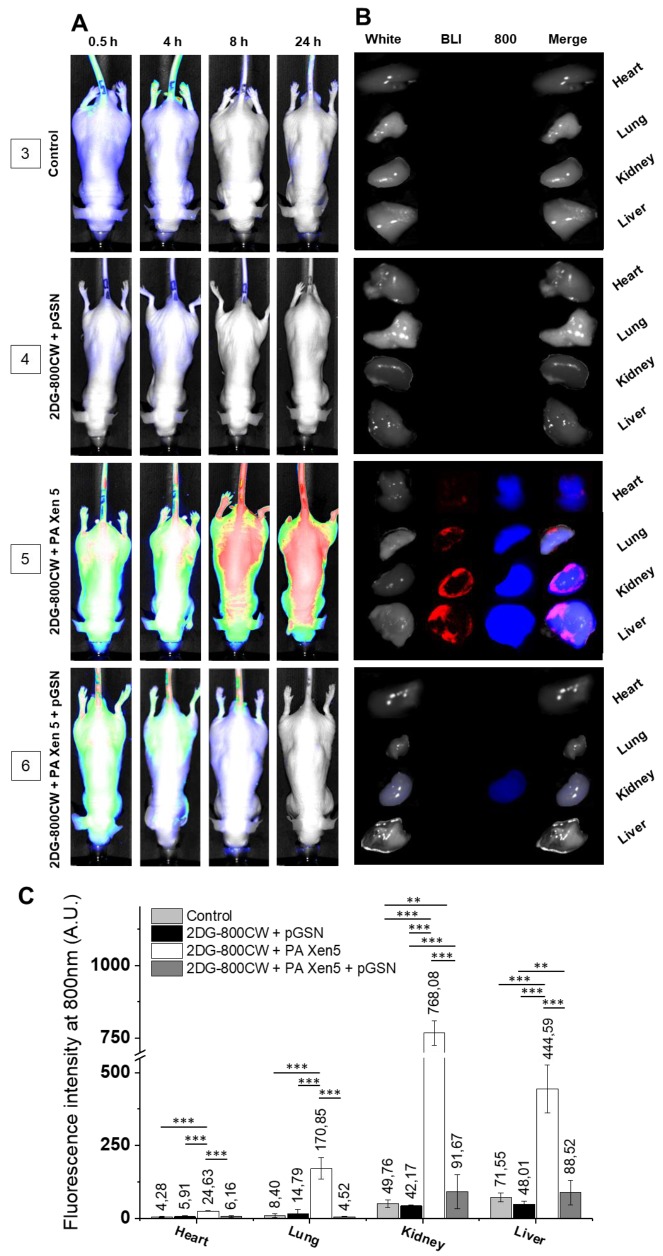
Visualization of systemic inflammation in vivo using analysis of IRDye^®^ 800CW 2-deoxy-D-glucose (2DG-800CW) fluorescence. 2DG-800CW was injected at the dose of 10 µM to non-infected, control animals (group 3); healthy animals were injected with pGSN at the dose of 40 mg/kg (group 4); PA Xen5-infected, non-treated animals (group 5) and PA Xen5-infected animals were treated with pGSN at the dose of 40 mg/kg (group 6). At the start of the experiment, mice were injected intravenously with sterile saline (groups 3 and 4) or PA Xen5 inoculum (groups 5 and 6). Eight hours later, 2DG-800 CW at the dose of 10 µM was injected to all tested animals, followed by administration of pGSN (40 mg/kg) to animals from groups 4 and 6. Data were collected after 0.5, 4, 8 and 24 h from the start of observation. Results from representative animals are shown (**A**). When animals were sacrificed (24 h after injection of 2DG-800CW), hearts, lung, kidney and livers were collected, and fluorescence signal was registered at the BLI and 800 nm channels. Results from representative organs are presented (**B**). To more quantitively estimate the level of inflammation in mouse organs, the fluorescence signal collected using an 800 nm channel (indicating the inflammation severity) was calculated and presented as mean value ± SD from three areas of each organ. ** and *** indicate statistical significance (*p* < 0.01 and *p* < 0.001, respectively; assessed using one-way ANOVA) when comparing to corresponding organs in mice of other tested groups (**C**).

The Pearl^®^ Trilogy small animal imaging system (Li-COR, Lincoln, NB, USA) used in this study allowed simultaneous monitoring of both inflammation burden and microbial infection development and severity (Figure 2). The signal from 2-deoxyglucose labeled with IRDye^®^800CW (2DG-800CW), the uptake of which is recognized as an indicator of inflammation [21], was tracked using the 800-nm channel, and the bioluminescent signal from *P. aeruginosa* Xen5 was collected using the bioluminescent (BLI) channel. In contrast to control healthy animals, where 2DG-800 CW was quickly and effectively eliminated (Figure 2A, group 3), in vivo imaging of PA Xen-5 mice (i.e., developing sepsis), showed dynamic changes in fluorescence signal intensity. At 24 h from the start of observation, a strong intensity signal for 2DG-800CW for all collected organs was recorded, which confirmed a systemic infection and inflammatory state with liver and kidneys as the most burdened with bacterial infection (Figure 2C). Importantly, the administration of exogenous pGSN to mice with PA Xen5-induced sepsis caused a rapid decrease in bacterial-derived chemiluminescence (Figure 2B, group 6). The signal from bacterial cells was also not observed during the analysis of organs from sacrificed animals, which indicates a sufficient anti-inflammatory effect. The residual signal was registered only for 2DG-800CW accumulated in kidneys, which most probably indicates the final phase of silencing inflammation (Figure 2B, group 6).

**Figure 3:**
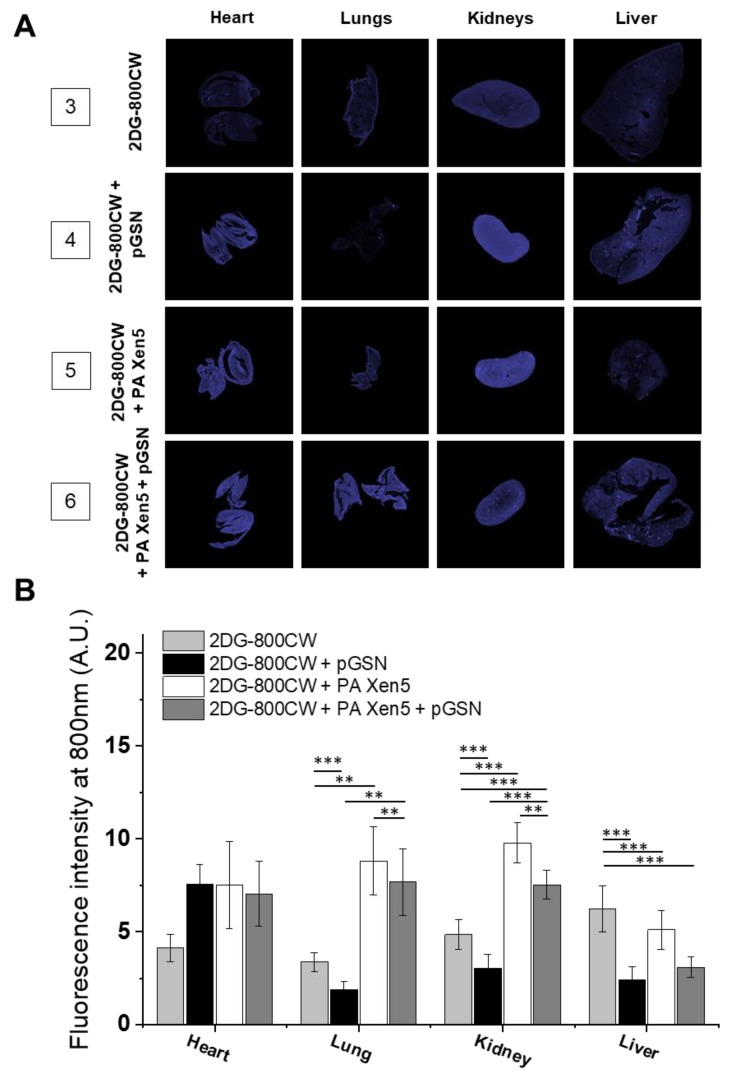
Estimation of inflammation burden in Cby.Cg-Foxn1nu/cmdb mice organs based on measurement of 2DG-800CW-targeted fluorescence intensity. At the start of the experiment mice were injected intravenously with sterile saline (groups 3 and 4) or PA Xen5 inoculum (groups 5 and 6). Eight hours later 2DG-800 CW at the dose of 10 µM was injected to all tested animals, followed by the administration of pGSN (40 mg/kg) to animals from groups 4 and 6. When animals were sacrificed (24 h after injection of 2DG-800CW), the hearts, lung, kidney and livers were collected, and the fluorescence signal was registered at the BLI and 800 nm channels. Results from representative animal organs are presented (**A**). To more quantitively estimate the level of inflammation in mice organs, the fluorescence signal collected using an 800 nm channel (indicating the inflammation severity) was calculated and presented as mean value ± SEM from three areas of each organ. ** and *** indicate statistical significance (*p* < 0.01 and *p* < 0.001, respectively; assessed using one-way ANOVA) when comparing to corresponding organs in mice of other tested groups (**B**).

The limitation of bacterial-induced inflammation was also confirmed by analysis of paraffin-embedded organs collected after autopsy of animals (Figure 3). Surprisingly, although—overall—we observed an anti-inflammatory effect of pGSN in the collected organs, some increase in 2DG-800 CW uptake was noted in the hearts of the pGSN-injected animals. Nevertheless, at this point we do not have any reports indicating the ability of pGSN to induce inflammation, and to suggest such an effect, more experimental data that are out of the scope of current manuscript should be acquired.

These observations agreed with the clinical scoring of animal behavior performed simultaneously with fluorescence-based observations. The comparison of clinical outcomes of PA Xen5-infected animals during sepsis and pGSN-treated mice indicated that administration of exogenous pGSN considerably improved the survival of animals (60% versus 100% in non-treated and treated groups, respectively). Notably, the appearance of pGSN-treated mice and their behavior did not differ from healthy ones that did not have induced sepsis as elucidated by both researchers and technical employees of Center for Experimental Medicine in Bialystok.

### 2.4. Histopathological and Biochemical Analysis

**Figure 4:**
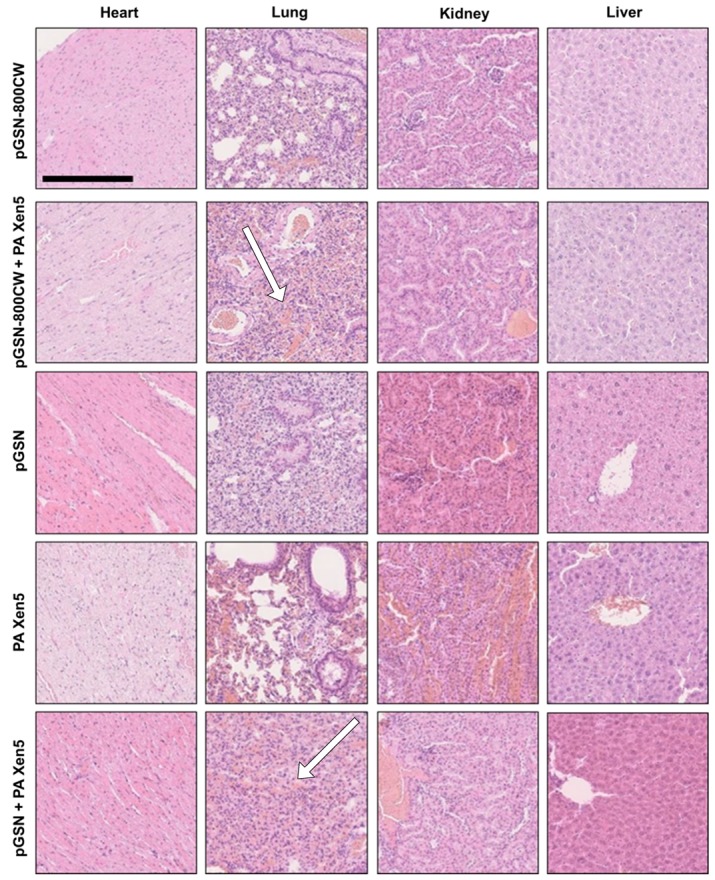
Histological analysis of the main organs of Cby.Cg-Foxn1nu/cmdb mice. Hematoxylin-eosin staining was performed to examine histopathological changes (200×). Lung injury was characterized by accumulation of blood (white arrows). Black scale bar indicates 250 µm. Results from one representative experiment are shown.

The summary of results obtained from hematoxylin-eosin staining of collected organs is presented in Table 2 and Figure 4. In all examined groups, heart morphology was normal, but some differences in morphology of other collected organs were recorded. The most prominent pathological changes, particularly hyperaemia, were noted for bacterial-infected, non-treated animals (group 5). Importantly, pGSN administration partially reversed PA Xen5-induced alterations in organs. In the pGSN-800 CW + PA Xen5 and pGSN groups, some hepatic cell degradation was noted.

As an additional analysis, histopathological evaluation of each animal’s pancreas was performed. According to results shown in Figure S2, acute interstitial edematous, focal necrosis and necrosis in the pancreas were noted. However, this observation was not reproduced in our other studies using Wistar rats as tested animals [14].

**Figure 5:**
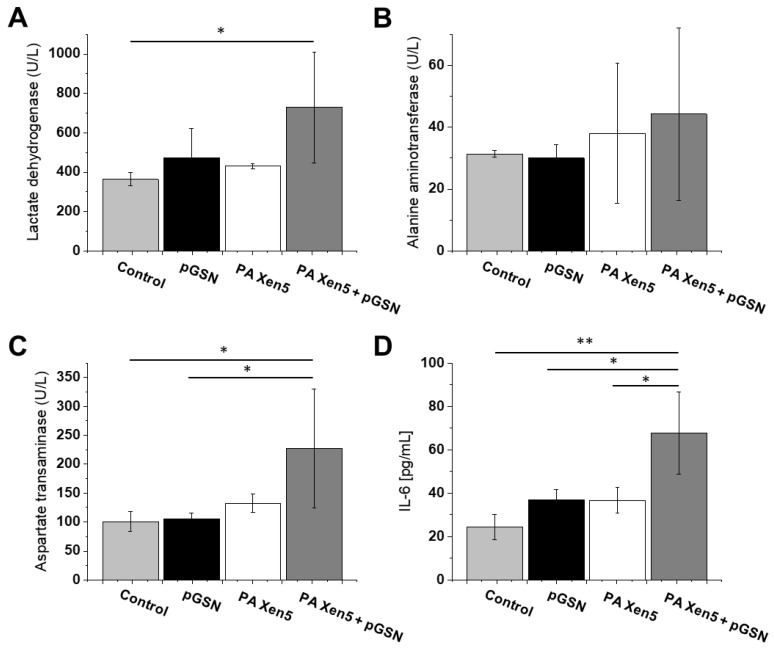
Blood plasma levels of LDH (**A**), ALT (**B**), AST (**C**) and IL-6 (**D**) recorded in blood samples collected from control, healthy Cby.Cg-Foxn1nu/cmdb mice (light grey columns; group 3, *n* = 5), healthy, pGSN-injected mice (black columns; group 4, *n* = 5), PA Xen 5-infected mice (white columns; group 5, *n* = 5) and pGSN-treated mice (dark grey columns; group 6, *n* = 5). At the start of the experiment mice were injected intravenously with sterile saline (group 3 and 4) or PA Xen5 inoculum (group 5 and 6). Eight hours later pGSN was added (40 mg/kg) to animals from groups 4 (pGSN, black columns) and 6 (PA Xen5 + pGSN, dark grey columns). After sacrificing the animals (24 h after injection of pGSN), blood was collected and tested for indicated markers. Results are presented as mean value ± SD. * and ** indicate statistical significance (*p* < 0.05 and *p* < 0.01, respectively; assessed using one-way ANOVA).

To elucidate whether alterations in histopathology of collected organs are mirrored in the biochemical profile in pGSN-treated animals, we performed analysis of lactase dehydrogenase (LDH), alanine aminotransferase (ALT) and aspartate transaminase (AST) concentration in the plasma of sacrificed animals. The level of IL-6 was also evaluated. Results are presented in Figure 5. As presented pGSN itself might induce LDH release and ALT and AST increase, although these differences did not reach statistical significance. A stronger response was detected in bacteria-infected animals treated with pGSN (group 6), and it might reflect the alterations in histopathology of collected livers. Regardless, those changes did not significantly impact the apparent health of animals, considering the overall clinical behavior of pGSN-treated animals and their improved survival, when compared to nontreated animals. In our biochemical analyses, only the rise of IL-6 concentration reached statistical significance. As shown in Figure 5D, treatment with exogenous pGSN increased the expression of IL-6 to 67.7 pg/mL compared to control and PA Xen5-infected animals (24.3 pg/mL and 36.6 pg/mL, respectively). Notably, pGSN itself is able to increase IL-6 concentration, which might be the reason for such an elevated level of this cytokine.

### 2.5. Bactericidal Activities of Exogenously Administrated pGSN

**Figure 6:**
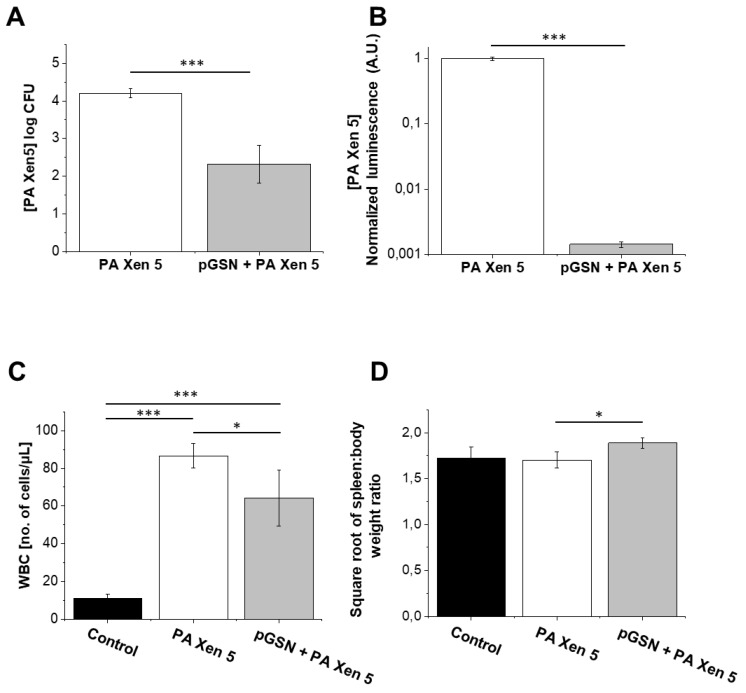
Decrease in bacterial outgrowth and colony forming capability of PA Xen 5 after pGSN treatment (40 mg/kg) (grey columns, group 6, *n* = 5) when compared to bacterial-infected untreated mice (white columns, group 5, *n* = 5) (**A**). Reduction of chemiluminescence signal of *P. aeruginosa* Xen 5 from abdominal fluid collected after pGSN treatment (40 mg/kg) (grey columns, group 6, *n* = 5) compared to bacterial-infected, untreated mice (white columns, group 5, *n* = 5) (**B**). Total amounts of WBCs in the blood of healthy mice (control; black columns, group 3, *n* = 5), infected mice (PA Xen 5; white columns, group 5, *n* = 5), and infected animals treated with pGSN (40 mg/kg) after *P. aeruginosa* Xen 5 challenge (pGSN + PA Xen 5; grey columns; group 6, *n* = 5) (**C**). Square root of the spleen weight/total body weight ratio recorded for organs collected from healthy (control; black columns, group 3, *n* = 5), infected (PA Xen 5; white columns, group 5, *n* = 5) and pGSN-treated (40 mg/kg) mice (pGSN + PA Xen 5; grey columns, group 6, *n* = 5) (**D**). At the start of the experiments, animals from the above groups received i.v. sterile saline (group 3) or PA Xen5 inoculum (group 5 and 6). Eight hours later, animals were injected with pGSN (40 mg/kg) (group 6) or left untreated (group 5). Peritoneal fluid, blood and spleen were collected from sacrificed animals 24 h after the start of pGSN treatment (40 mg/kg). Results are presented as mean value ± SD. * and *** indicate statistical significance (*p* < 0.05 and *p* < 0.001, respectively; assessed using two-tailed Student’s *t*-test (**A**,**B**) or one-way ANOVA (**C**,**D**). Analysis was made using Cby.Cg-Foxn1nu/cmdb mice.

As presented in Figure 6, pGSN was effective in significantly reducing bacterial outgrowth (CFU) from peritoneal fluid samples collected after autopsy of septic mice. The administration of pGSN caused a two log decrease in PA Xen 5 outgrowth in nutrient media (*p* = 0.0.31; Figure 6A) and chemiluminescence in peritoneal fluid was 500-fold smaller in pGSN-treated infected mice when compared to Pa Xen5-infected but untreated animals (*p* < 0.001; Figure 6B). In agreement with that result, total WBC levels decreased for the pGSN-treated group compared to the bacteria-infected animals (Figure 6C). As shown in Figure 6D, slight differences in the spleen weight/total body weight ratio among animal groups were observed.

### 2.6. pGSN Increases Phagocytosis of PA Xen5 by Macrophages

**Figure 7:**
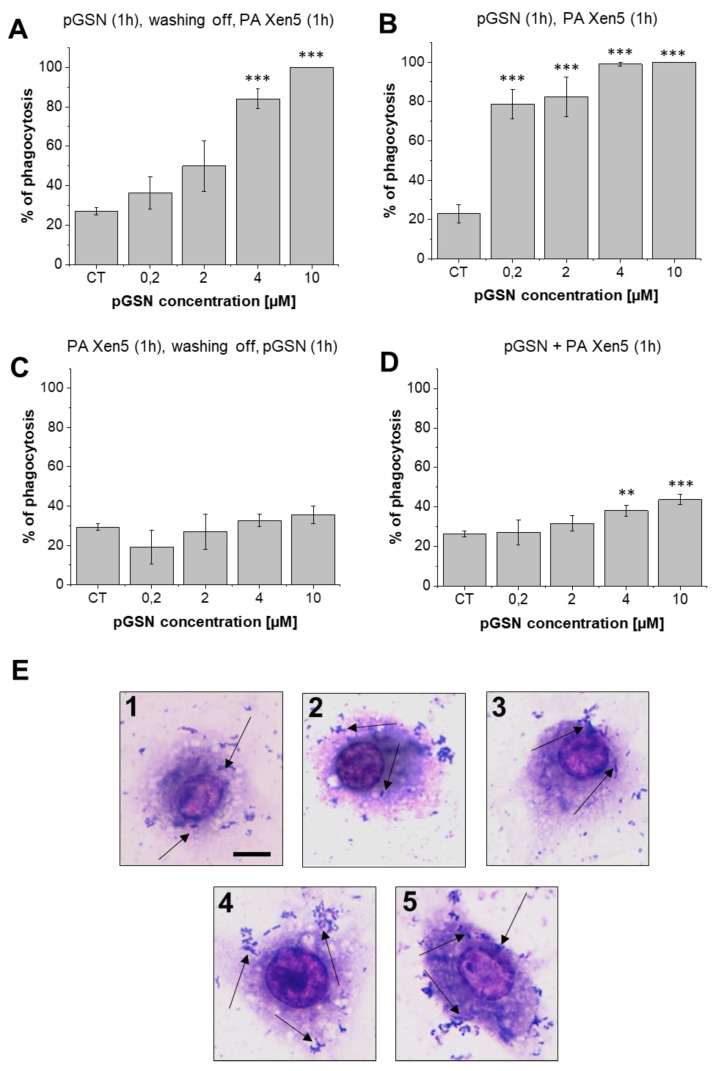
pGSN-mediated stimulation of phagocytosis by RAW264.7 in in vitro settings. Effect of exogenous pGSN on internalization of PA Xen 5 by macrophages, when administrated in varied combinations with bacterial suspension: pGSN (0.2–10 µM) incubation for 1 h, washing off and PA Xen 5 addition for 1 h (**A**), pGSN (0.2–10 µM) incubation for 1 h followed by the addition of PA Xen 5 without washing off of pGSN (**B**), macrophage incubation with PA Xen 5 for 1 h, washing off and pGSN addition for 1 h (0.2–10 µM) (**C**) and a simultaneous incubation of macrophages with both pGSN (0.2–10 µM) and bacteria for 1 h (**D**). Results are presented as mean value ± SD from three individual experiments. ** and *** indicate statistical significance (*p* < 0.01 and *p* < 0.001, respectively; assessed using two-tailed Student’s *t*-test) when compared to non-stimulated control cells (CT). Microscopic analysis of phagocytosis of *P. aeruginosa* Xen 5 by macrophages RAW 264.7 (**E**). Macrophages on collagen type I-coated cover glasses were cultured with PA Xen 5 for 1 h with (1) 0 µM, (2) 0.2 µM, (3) 2 µM, (4) 4 µM and (5) 10 µM of pGSN and stained with Giemsa. Black arrows indicate *P. aeruginosa* Xen5 bacteria. Black scale bar indicates 10 µm.

As presented in Figure 7A, a 1 h preincubation of macrophages with pGSN followed by washing off and addition of *P. aeruginosa* Xen5 resulted in a dose-dependent increase in the level of phagocytosing cells (nearly 5-fold increase at a dose of 10 µM when compared to control cells). Simultaneously, 1-h preincubation of RAW264.7 with pGSN and further incubation of macrophages with both pGSN with PA Xen 5 increased the number of phagocytic macrophages to over 80%, even at very low gelsolin concentrations (0.2 and 2 µM) (Figure 7B). This effect was not so prominently observed when macrophages were cultured first with bacteria (Figure 7C) or treated simultaneously with both pGSN and PA Xen5 (Figure 7D,E).

## 3. Discussion

Sepsis and septic shock represent very serious medical conditions that require a complex therapeutic approach and use of a spectrum of drugs assuring multidirectional biological activity. A compelling number of studies report that pGSN, apart from having actin-scavenging properties, exerts a number of effects in pathogenesis including protection against infections, implication in wound healing and re-epithelization, bone remodeling, and the stabilization of intracellular calcium concentrations [5,22]. Importantly, in mice not expressing GSN, dysfunction in inflammatory reactions, including the delayed in vivo migration of neutrophils and impaired chemotaxis was observed [23].

In this study we aim to evaluate the anti-inflammatory and immunomodulatory effects of exogenously administrated pGSN in a mouse model of sepsis. Considering that a great majority of opportunistic and hospital-acquired infections, including sepsis, occurs in patients with weakened host defense mechanisms, we employed in this study the Cby.Cg-Foxn1nu/cmdb mouse strain, characterized by impaired immune response, which in our opinion best reflects the clinical picture of patients in the course of the sepsis. Real-time observations using fluorescence-conjugated pGSN assessed the biodistribution and organ uptake of injected i.v. pGSN, which has been relatively unexplored. We observed a relatively high accumulation of pGSN in liver and kidneys, particularly in bacterial-infected mice (Figure 1), which would be in agreement with previous reports using radioactive-labeled plasma gelsolin to evaluate its kinetics in rabbits [24]. Lind et al. demonstrated that (i) liver is the main site of accumulation and degradation of plasma gelsolin and (ii) kinetic parameters obtained from rabbit-based analyses are comparable to those obtained for human pGSN, which highlights the possibility to translate these observations to human conditions. Notably, pGSN clearance from the circulation was accelerated by actin [24], which might explain the increased fluorescence intensities recorded for kidneys in PA Xen 5-infected animals. In previous reports, significantly lowered blood levels of pGSN (~50% of controls) were noted in adult mice subjected to lethal doses of LPS or CLP assault, which was presumably determined by the release of actin [12]. The administration of exogenous pGSN improved the survival and clinical outcomes of pGSN-treated mice, most probably through combined mechanisms, involving (i) the sequestering of free actin released from the damaged cells, (ii) the binding of inflammatory mediators and neutralization of their pro-stimulatory effects, and (iii) the limitation of acute inflammatory reactions due to alterations in cytokine expression [12,14]. The route of exogenous gelsolin administration has been reported to determine the therapeutic efficiency of pGSN, since CLP-induced sepsis in rats was inhibited by subcutaneous and intravenous, but not intraperitoneal injection of plasma gelsolin, which strongly indicates the necessity of systemic action of pGSN, in order to improve the clinical outcomes of septic subjects [14]. In agreement with these reports, we show that intravenous administration of exogenous pGSN decreases the inflammatory response in sepsis that develops as a result of *P. aeruginosa* Xen5 intraperitoneal injection (Figure 2). Simultaneously, by monitoring the BLI channel we demonstrated that dynamic bacterial-derived fluorescence intensity was limited in gelsolin-treated mice, which indicates decreased infection followed by bacterial eradication (Figure 2A,B, group 6). In vivo observations were validated by ex vivo fluorescence imaging of organ sections (Figure 3). Analyses using near-infrared labels and fluorescence-based probes allowed the investigation of the overall and whole body inflammation state, and not only examination of specific, single parameters of infection and inflammation (particularly WBC or cytokine levels [14]), in which lies the novelty of this research. Based on the presented results, this study confirms that plasma gelsolin serves as a systemic anti-inflammatory and immunomodulatory compound with the ability to limit whole body inflammation, regardless of the type and location of the tissue in which the inflammation develops and from which the infection originates. Overall, this molecular shield might be crucial for surviving the medical condition associated with hypogelsolinemia. Moreover, pGSN-mediated treatment was shown to improve the clinical morphology of organs collected from PA Xen5-infected animals (Figure 4). Even though some pGSN-induced alterations in organ morphology occur, particularly in the liver and pancreas, the improved clinical status and the survival ratio of pGSN-treated animals suggest that this effect is not overwhelming. Considering that some changes recorded in the histopathology of pancreas (Figure S2) were not reproduced in Wistar rats [14], we suggest that this phenomenon is strain-specific. Moreover, to date, evidence of pancreatic injury has not been observed in human subjects, which confirms the safety and relatively low toxicity of pGSN-mediated therapy.

An important observation noted during this study was the decrease in bacterial outgrowth and bacterial-derived chemiluminescence from the peritoneal fluid of sacrificed, pGSN-treated animals (Figure 6A,B). Our findings are consistent with previous observations indicating gelsolin anti-LPS activity [12] and provide evidence of additional gelsolin properties that might result in the improvement of antimicrobial host defense. Accordingly, we propose that the anti-septic features of pGSN should be expanded by the ability of plasma gelsolin to limit bacterial multiplication and thus to reduce the microbial-induced inflammatory effects. The results shown in Figure 7 encourage the hypothesis that pGSN exerts an antimicrobial effect by reinforcing host immune defenses, since the preincubation of macrophages with pGSN significantly increased the number of cells that internalized bacteria. It would be in agreement with previous reports demonstrating a role for intracellular gelsolin in modulation of Fc-receptor and integrin-mediated phagocytosis [25,26]. Considering the recent data about the implication of phagosomes in antigen presentation processes [27], it might also be hypothesized that pGSN, due to the stimulation of phagocytosis, supports further steps of adaptive immune mechanisms, acting as a link between innate and adaptive antimicrobial mechanisms.

Previous reports support the hypothesis that plasma gelsolin, in a manner analogous to LPS-binding protein (LBP), might act both as an endotoxin-binding factor and LPS-presenting protein, depending on its plasma concentrations and the LPS/pGSN ratio. Although originally LBP was recognized as a key factor triggering the inflammatory response to microbial assault [28], further analyses using animal models of sepsis demonstrated that exogenously administrated recombinant LBP is able to decrease LPS-induced cytokine release and improve the clinical outcomes of intraperitoneally infected animals [29,30], which could be explained by LBP acting as bacterial phagocytosis enhancer [31,32]. This issue should be investigated in more complex experimental models. Similarly, the contradictory reports about LBP-mediated activities encourage the hypothesis that the biphasic action of gelsolin depends on cell types, pGSN concentration and LPS/pGSN ratio. Particularly, the effect of pGSN on cytokine release needs more clarification, since both an increase in and limitation of pro-inflammatory cytokines, including TNF-α and IL-6, have been reported [14]. In our experimental settings, a slight increase in IL-6 level was noted (Figure 5D), but considering the clinical picture of pGSN-treated animals, and the improvement of behavior, survival and clinical outcomes of treated animals, this increase might reflect reinforcement of immune responses against bacterial assault that promote beneficial suppression of LPS-induced effects in analogy to reactive oxygen species, which in decreased concentrations are responsible for impaired physiological functions, but at higher concentrations cause oxidative stress and irreparable DNA damages. Similarly, we suggest that a slight pGSN-mediated increase in IL-6 production might reflect the return of this factor to its physiological balance resulting in improvement of clinical outcomes in inflammatory state.

## 4. Materials and Methods

### 4.1. Labeling of pGSN with IRDye^®^ 800CW

Recombinant human plasma gelsolin (pGSN) used in our study was produced in *E.coli* and provided by BioAegis Therapeutics (North Brunswick, NJ, USA). Conjugation of pGSN with IRDye^®^ 800CW was performed using the IRDye^®^ 800CW Protein Labeling Kit (Li-Cor Biosenses, Lincoln, NB, USA), according to manufacturer guidelines. In order to confirm the proper conjugation of pGSN with IRDye^®^ 800CW (pGSN-800CW), physicochemical properties of pGSN before and after labeling reaction were assessed, using Fourier transform infrared spectroscopy (FTIR), differential scanning calorimetry (DSC) and thermogravimetric analysis (TGA).

### 4.2. Induction of Sepsis due to Peritonitis

Ten-week-old nude female mice (strain Cby.Cg-Foxn1nu/cmdb; imported from The Jackson Laboratory (Bar Harbor, ME USA) weighing 20–25 g, were used for this study, in accordance with protocols approved by the Local Ethic Committee in Bialystok of the Medical University of Bialystok no 102/2015. Murine peritonitis was established as described previously [33] using *P. aeruginosa* Xen5, a bioluminescent strain possessing a stable copy of the *Photorhabdus luminescens* lux operon on the bacterial chromosome (Perkin Elmer, Waltham, MA USA). Briefly, broth cultures of freshly plated *P. aeruginosa* Xen5 bacteria were grown to logarithmic phase overnight to an optical density of 0.5 at 600 nm, centrifuged, purified from the culture medium, and adjusted with sterile saline solution immediately, before experiments to OD_600_ of 0.15, which corresponds to 6 × 10^6^ CFU/mL. In order to induce sepsis, randomly assigned mice were injected intraperitoneally with 100 μL of bacterial suspension (*n* = 5) or sterile NaCl 0.9% as a control (*n* = 5), under sterile conditions. Mice were left under observation for 8 h to allow peritonitis-induced sepsis to develop, and all further procedures were performed 24 h after bacterial/saline injection. All infected animals became quieter than usual, with less movement, apathy and behaviorally exhibited signs of septic infection. The survival rates of mice were monitored for up to 32 h after peritonitis induction.

### 4.3. Biodistribution of pGSN-800CW

An amount of 40 mg/kg pGSN in a volume of 100 µL was injected intravenously into non-infected (*n* = 5) and PA Xen5-infected (*n* = 5) mice 8 h later. At 24 h post-injection, a lethal injection of xylazine-ketamine was administered, and then the heart, lungs, kidneys and liver were collected. Removed organs were fixed in 4% paraformaldehyde, paraffin embedded, and sectioned. The fluorescence of isolated organs was recorded in the 800-nm channel.

### 4.4. Evaluation of Anti-Inflammatory and Bactericidal Effects of pGSN Administration. Histopathological Analysis

Under sterile conditions, nude mice were divided into control (*n* = 5), PA Xen5-infected (*n* = 5; with developed sepsis) and pGSN-treated (*n* = 5; with sepsis and further treated with pGSN) groups. A near-infrared fluorescence labeled 2-deoxyglucose analog, IRDye^®^ 800CW 2-deoxyglucose optical probe (2DG-800CW) [21], was employed as an indicator of inflammation severity and injected intravenously to control, untreated and treated mice at a volume of 100 µL. IRDye 800CW-labeled 2-deoxyglucose was administrated in a final dose of 10nM, according to producers guidelines. Injection of 2-DG 800CW was performed 8 h after *Pseudomonas aeruginosa* Xen5 injection, i.e., at the start of pGSN treatment. At indicated time points after the injection of pGSN, animals were anesthetized, and mice were scanned at 800 nm for the targeted 2DG-800CW fluorescence signal.

To evaluate the immunomodulatory activity of exogenously administrated pGSN, 40 mg/kg of pGSN in a volume of 100 µL was injected intravenously 8 h post bacterial challenge. Control and untreated animals obtained i.v. 100 µL of saline, as vehicle control. At predetermined times (0.5, 4, 8 and 24 h post pGSN administration), mice from all groups were anesthetized with isoflurane, and whole-body scans were performed. After 24 h, a lethal injection of xylazine-ketamine was administered, and the heart, lungs, kidneys, and liver were isolated from all animals. Collected organs were scanned using the bioluminescence channel (BLI; for assessment of PA Xen5 burden) and the 800-nm channel and then fixed in 4% paraformaldehyde, paraffin embedded and sectioned. The spleens were removed and the weight was measured to assess possible change due to inflammation. After an autopsy, peritoneal fluid and blood were collected for further analysis. Peritoneal fluid was spotted on cetrimide-containing agar plates for overnight culture to determine PA Xen5 bacterial outgrowth and bacterial-derived the chemiluminescence signal in peritoneal fluid was also assessed. Blood collected by cardiac puncture was analyzed for total white blood cell (WBC) level. The tissues obtained from the experiments were fixed in 4% buffered formalin, and histopathological analysis using hematoxylin-eosin staining was performed.

As an additional indicator of the therapeutic efficiency of gelsolin-based treatment, the overall behavioral impression analysis of all mice was made. The assessment was performed by the persons participating in the experiment and an additional person who did not participate in the experiment.

### 4.5. Analysis of Phagocytosis Using In Vitro Settings

To assess gelsolin’s ability to modulate phagocytosis, mouse macrophage RAW264.7 were seeded on round 12-mm collagen type I-coated cover glasses and cultured in 24-well plates (RPMI 1640 medium, 5% CO_2_, 37 °C) until confluent monolayers were formed. Plasma gelsolin (0.2–10 µM) and bacteria (5 × 10^5^ CFU/mL) were added to cell cultures to obtain the ratio of bacteria to macrophages as 10:1 in varied combinations as follows: (i) pGSN incubation for 1 h, wash off and PA Xen5 addition, (ii) pGSN incubation for 1 h followed by addition of PA Xen5, (iii) macrophage incubation with PA Xen5 for 1 h, washing off and pGSN addition for 1 h and (iv) a simultaneous incubation of macrophages with both pGSN and bacteria for 1 h. Next, pGSN and/or PA Xen 5-treated cells were washed with PBS, fixed with methanol for 10 min, and stained using Giemsa. Phagocytosis of PA Xen5 by macrophages RAW 264.7 was observed by light microscopy. To quantitatively evaluate the pGSN-mediated effect, macrophages seen in 10 randomly selected fields of view were counted up to 100, and from this pool, phagocytic macrophages were calculated as a percentage of the total pool of cells.

### 4.6. Biochemical Analysis

The serum levels of lactate dehydrogenase (LDH) and IL-6 were measured using commercially available colorimetric kits (LDHL cobas C tests, Roche Diagnostics, Mannheim, Germany and Mouse IL-6 Quantikine ELISA Kit, R&D Systems, Minneapolis, MN, USA, respectively) following the manufacturer’s instructions. Plasma enzyme aspartate transaminase (AST) and alanine aminotransferase (ALT) activity were measured using Immunoanalyzer corbas e 411 (Roche Diagnostics, Mannheim, Germany)

### 4.7. Statistical Analysis

Significance of differences was determined using the two-tailed Student’s *t*-test or one-way ANOVA. Statistical analyses were performed using OriginPro 2020 (OriginLab Corporation, Northampton, MA, USA). *p* < 0.05 was considered to be statistically significant.

## 5. Conclusions

In summary, the experiments described here demonstrate the protective effects of exogenously administrated plasma gelsolin against bacteria-induced sepsis, which is determined by the immunomodulatory and anti-inflammatory activities of this compound and its ability to reinforce immune response mechanisms, including phagocytosis.

## Figures and Tables

**Table 1:** Groups of animals used in the study.

	No.	Description	No. of Animals	0 h	8 h	24 h
**Biodistribution**	**1**	pGSN-800CW	*n* = 5	Sterile saline	pGSN-800CW (40 mg/kg)	Collection of blood, peritoneal fluid and organs for analysis
	**2**	pGSN-800CW + PA Xen5	*n* = 5	PA Xen5 inoculum (6 × 10^5^ CFU/mL)	pGSN-800CW (40 mg/kg)	
**Anti-inflammatory**	**3**	Control	*n* = 5	Sterile saline	2DG-800 CW (10 µM)	
	**4**	2DG-800CW + pGSN	*n* = 5	Sterile saline	2DG-800 CW (10 µM) + pGSN (40 mg/kg)	
	**5**	2DG-800CW + PA Xen5	*n* = 5	PA Xen5 inoculum (6 × 10^5^ CFU/mL)	2DG-800 CW (10 µM)	
	**6**	2DG-800CW + PA Xen5 + pGSN	*n* = 5	PA Xen5 inoculum (6 × 10^5^ CFU/mL)	2DG-800 CW + pGSN (40 mg/kg	

**Table 2:** Histopathological analysis of organs collected from sacrificed PA Xen5-infected and pGSN-treated animals performed using hematoxylin-eosin staining.

	Heart	Lungs	Kidneys	Liver
**pGSN-800CW**	no change	slight atelectasis, few interstitial lymphocytes	no change	no change
**pGSN-800CW + PA Xen5**	no change	atelectasis *, hyperemia in the intestinal tissue	no change	slight hyperaemia, hepatic cell degradation, few lymphocytes in the liver sinuses
**pGSN**	no change	slight atelectasis, few interstitial lymphocytes	slight hyperaemia, few interstitial lymphocytes	hepatic cells degradation, few interstitial lymphocytes
**PA Xen5**	no change	slight atelectasis, hyperaemia *	hyperaemia *	hyperaemia *
**pGSN + PA Xen5**	no change	atelectatic changes *, hyperemia in the intestinal tissue	slight hyperaemia, few interstitial lymphocytes	slight hyperaemia

* indicates the most prominent changes when compared to specimens from other groups.

## Supplementary Materials

Supplementary materials can be found at https://www.mdpi.com/1422-0067/21/7/2551/s1.

## Abbreviations

2DG-800CW	IRDye 800CW-labelled 2-deoxyglucose
GSN	gelsolin
pGSN	plasma gelsolin
pGSN-800CW	IRDye 800CW-labelled plasma gelsolin
Pa Xen5	*Pseudomonas aeruginosa* Xen5 strain
