# Supplementary material for: Recombinant Human Plasma Gelsolin Stimulates Phagocytosis while Diminishing Excessive Inflammatory Responses in Mice with Pseudomonas aeruginosa Sepsis
Source: Int J Mol Sci. 2020 Apr 7;21(7):2551. doi: 10.3390/ijms21072551 (PMC7177774; doi:10.3390/ijms21072551)

Recombinant human plasma gelsolin stimulates phagocytosis while diminishing excessive inflammatory responses in mice with Pseudomonas aeruginosa sepsis

Ewelina Piktel, Urszula Wnorowska, Mateusz Cieśluk, Piotr Deptuła, Suhanya V. Prasad, Grzegorz Król, Bonita Durnaś, Andrzej Namiot, Karolina H. Markiewicz, Katarzyna Niemirowicz-Laskowska, Agnieszka Z. Wilczewska, Paul A. Janmey, Joanna Reszeć, Robert Bucki*

Supplementary material.

List of figures.

Supplementary Figure 1. Physicochemical properties of pGSN labeled by IRDye® 800CW (pGSN-800CW). FTIR spectra of unlabeled human plasma gelsolin (pGSN; grey line) and pGSN labeled by IRDye® 800CW (pGSN-800CW; green line) (panel A). Panels B-D show thermal properties of unlabeled pGSN (grey line) and pGSN labeled by IRDye® 800CW (pGSN-800CW; green line) evaluated using differential scanning calorimetry (panel B) and thermogravimetric analysis (panels C, D).

Supplementary Figure 2. Histological analysis of pancreas of Cby.Cg-Foxn1nu/cmdbmice. Hematoxylin-eosin staining was performed (50x and 200x). The presence of acute interstitial edematous (A), focal necrosis (B,C) and necrosis (D,E,F) in analyzed pancreas was noted.

Supplementary Figure 1

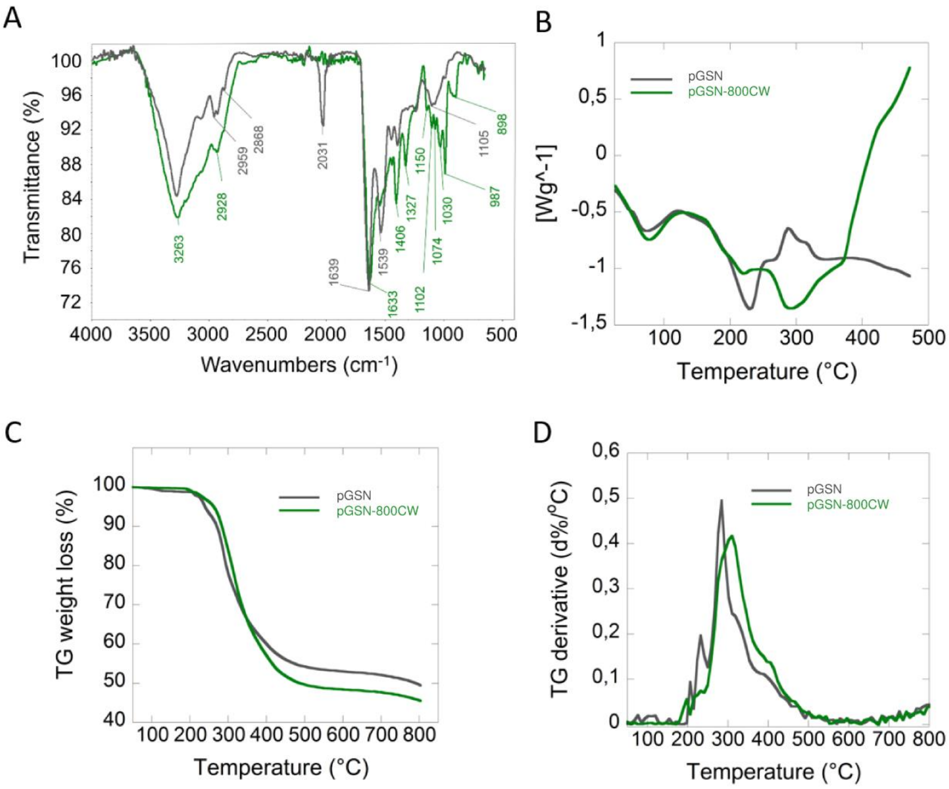

Supplementary Figure 2

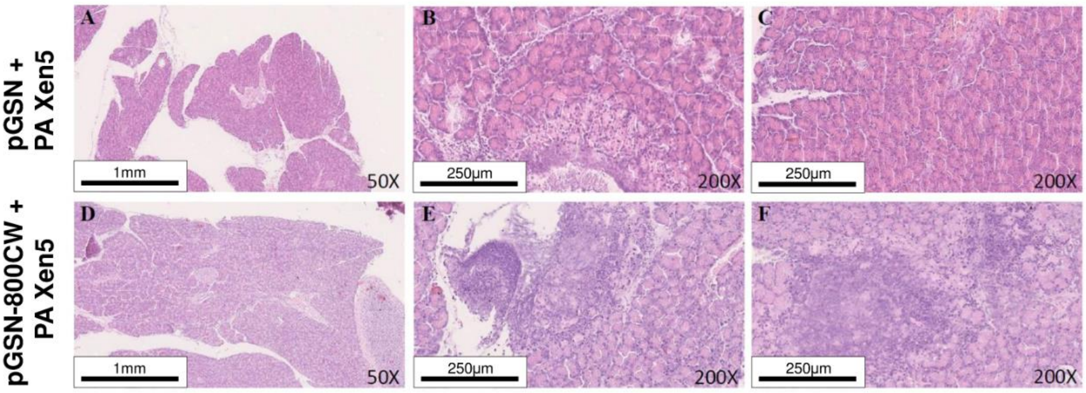

Supplement: Supplementary file 1 [file ijms-21-02551-s001.pdf]
